# Cellulose Nanofibrils Dewatered with Poly(Lactic Acid) for Improved Bio-Polymer Nanocomposite Processing

**DOI:** 10.3390/nano14171419

**Published:** 2024-08-30

**Authors:** Alexander Collins, Mehdi Tajvidi

**Affiliations:** School of Forest Resources, University of Maine, Orono, ME 04469, USA; awillcollins@gmail.com

**Keywords:** cellulose, biocomposites, polymers, sustainability, nanotechnology

## Abstract

Cellulose nanofibrils (CNFs) have theoretically ideal properties for bio-based composite applications; however, the incorporation of these materials into polymers is made challenging by the strong binding of water to CNFs and the irreversible agglomeration of CNFs during drying. Previous methods used “contact dewatering”, wherein the addition of wood flour (WF) to CNFs facilitated the mechanical removal of water from the system via cold pressing, which showed potential in producing dried CNF fibrils attached to wood fibers for biocomposite applications. In this work, the viability of contact dewatering with poly(lactic) acid (PLA) powder for PLA/CNF composites was evaluated. The energy efficiency of dewatering, preservation of nanoscale CNF morphology, and mechanical properties were examined by mixing wet CNFs with pulverized PLA at various loading levels, pressing water out of the system, and compression molding and shear mixing composites for testing. The most impressive results from this dewatering method were the preservation of micron-to-nanoscale fibers with high aspect ratios in PLA-CNF composites; increased strength and modulus of 1.7% and 4.2%, respectively, compared to neat PLA; equivalent or better properties than spray-dried nanocellulose at similar loading levels; and an 11-194x reduction in drying energy compared to spray-drying CNFs.

## 1. Introduction

Petroleum-based polymers represent a major concern for environmental impacts [1,2] due to CO_2_ emissions from petroleum sourcing and utilization, their slow degradation in natural environments, and the contamination of marine environments and soils with potentially hazardous breakdown products such as microplastics. The dwindling supply of oil reserves required to produce these polymers [3] further necessitates bio-based, sustainable replacements for polymers in the manufacturing economy. Cellulose nanofibrils (CNFs), the fundamental structural elements of plants, have shown potential for consumer product applications, attributed to their high theoretical mechanical properties [4,5], inherent biodegradability due to natural hydrolysis [6], low environmental or human health and safety risks [7], and potential to be manufactured with existing paper pulping infrastructure and equipment with slight modifications [8]. If used as a reinforcement material for polymers from renewable resources, such as poly (lactic) acid (PLA), CNFs have promise as a material for making fully compostable natural fiber–plastic composites for consumer, construction, aerospace, and automotive applications.

The use of CNFs in natural fiber–plastic composites, however, is complicated by the irreversible agglomeration of CNFs during drying, which reduces the aspect ratios of the fibers and, therefore, their practicality for reinforcing plastic composites [9]. Since CNFs are heavily refined paper pulp, water removal from CNF slurries presents issues for energy efficiency and compatibility with hydrophobic polymer matrices. No universal drying methods sufficiently preserve the nanoscale structure of CNFs or fibril length/width aspect ratios after drying [10]. Spray-drying, a process that can consistently produce dried powders from CNFs, has a high energy cost with a low production rate and does not preserve fibrillar aspect ratios, instead producing micron-scale agglomerates [11].

Previous experiments have demonstrated that the addition of wood particles to CNFs allows for the retention of some nanoscale structures of CNFs after freeze-drying or oven-drying [12,13], which has been attributed to “contact dewatering”. In contact dewatering, the addition of wood flour (WF) provided favorable surface interactions between CNFs and WF that facilitated water removal from the system via mechanical pressing [14,15]. Previous work from this research group examined contact dewatering of CNFs and lignin-containing CNFs (LCNFs) with maple wood flour, followed by drying and incorporating the hybrid furnishes into PLA/WF/CNF composites. This body of work demonstrated that observable nanoscale CNFs can be preserved on the surface of WF after drying, and this method has the potential to reduce the energy impacts of drying CNFs due to the removal of free water from the system in cold pressing. The incorporation of WF-CNFs into the final composites, however, did not strongly improve the strengths or moduli compared to neat PLA, with the best performing PLA/WF/CNF in one study composite showing equivalent tensile strength and a 6% improvement in tensile modulus compared to PLA/WF [16].

Because of potential interfacial issues caused by loading hybrid WF-CNF furnish into PLA composites and to eliminate WF as a CNF carrier, the effect of directly dewatering CNF with PLA particles was examined in this work. Numerous previous methods have explored directly incorporating CNFs into PLA nanocomposites, including solvent-exchanging or acetylating CNFs and mixing with dissolved PLA [17,18], or mixing PLA with spray-dried CNFs (SDCNFs) [19], but none dewatered CNFs directly onto PLA particles. This work was influenced by polymer latex-processed CNFs, in which polymer latex micelles separate and disperse CNF fibrils during liquid extraction, preventing them from agglomerating [20,21]. The latex processing concept was not previously explored using solid polymer particles to remove water from CNF suspensions or create dry thermoplastic mixtures for compounding, and it has not been determined if adequate CNF dispersion can be achieved in this way. The goal of this work was to develop an efficient method for dewatering, drying, and incorporating CNFs into functional PLA-CNF nanocomposites without the use of noxious solvents or spray-drying methods, just using the physical interactions between PLA and CNFs during the dewatering process. Calculations to determine the minimum level of CNF to produce a monolayer to cover all PLA particles in a mixture are included to understand how to optimize the system to produce small-scale CNFs attached to the surface of PLA. 

## 2. Materials and Methods

### 2.1. Manufacturing and Characterization of Mixtures

Cellulose nanofibrils (90% fines, ~3 wt.% solids) derived from bleached softwood kraft pulp were sourced from the UMaine Process Development Center. Amorphous PLA pellets (Natureworks Ingeo 3D700, Minnetonka, MN, USA) were shipped to Advanced Cryogenic Enterprises (Akron, OH, USA) and cryo-milled to produce cryocrushed PLA powder.

**Figure 1:**
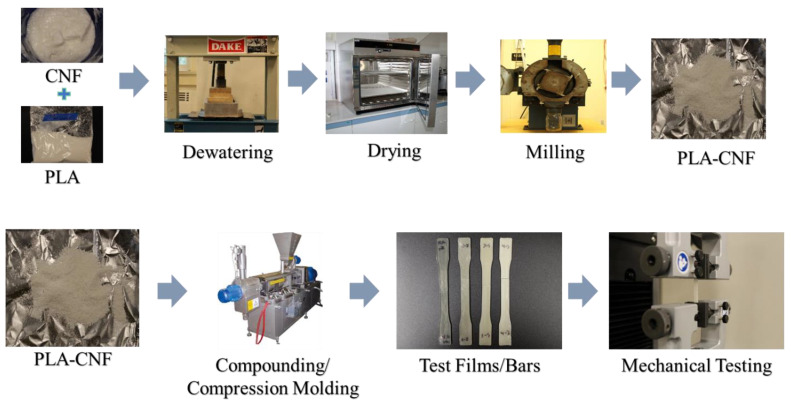
Outline for PLA-CNF dewatering and manufacture of composites.

The process for composite manufacture and testing is outlined in Figure 1. Powdered PLA and CNFs were combined at 10, 30, and 50 wt.% CNF loading levels, with 5 wt.% cumulative solids mass targeted in each mixture. To make each mixture, a calculated mass of water was first added to the CNFs, then the desired mass of powdered PLA was mixed into the suspension. The mixture was homogenized in a KitchenAid Artisan 5 stand mixer (St. Joseph, MI, USA, power level 1, 3 min). One gram was sampled from each mixture for moisture content analysis (Ohaus MB45) to determine solids content before pressing. Dewatering was performed on 100–150 g of each mixture by pressing in a DAKE (Grand Haven, MI) press setup. A square aluminum retainer was used to contain the material during press events, with a targeted ~2.2 MPa (over ~410 cm^2^ press area) of pressure. Meshed metal screens were placed underneath and on top of mixtures to better distribute pressure during dewatering, and paper towels under the pressing apparatus were used to absorb excess water and prevent re-absorption by the mixture. For film manufacture, PLA and CNFs were combined at lower loading levels (0.1, 0.5, 1, 2, 6, and 10 wt.% CNFs in PLA powder) and homogenized in a JJ-1 overhead mixer (VWR, Radnor, PA, USA), then pressed for dewatering. 20 wt.% was targeted as the solids content for these mixtures. This process was also used to make larger quantities of PLA-CNF mixtures for dewatered shear-mixed samples (0.5, 1, and 2 wt.% CNFs).

The wet dewatered materials (up to 50 wt.% initial mix solids) were processed into smaller fractions in the KitchenAid mixer, then dried at 50 °C in a Fisherbrand oven (Fischer Scientific, Waltham, MA, USA) for 16 h. The dewatered cakes for materials pressed at 20 wt.% solids (0.5–2 wt.% CNF) were placed in the oven directly after pressing to keep the cake structure preserved, then dried at 50 °C for 16 h. The cake structure was preserved to maintain the dispersion of CNFs achieved during the dewatering process and prevent CNF-CNF interactions during drying. After drying, materials were homogenized using a Wiley Mill (Thomas Scientific, Swedesboro, NJ, USA) utilizing a 1 mm mesh sieve screen. Sieve analysis was used to examine if CNFs were attached to PLA particles and increased diameters. This was performed in a sieve shaker (Retsch, Newtown, PA, USA) with 500–150 µm sieve pan sizes. A 10 g sample per CNF loading level was sieved with an amplitude of 1 for 20 min, after which the mass per sieved particle diameter was determined by subtracting the known empty pan mass from the measured pan mass after sieving. The results were calculated in terms of mass per sieve size over the total particle mass of the sieved mixture. Scanning electron microscopy (SEM) was used to observe whether dry, dewatered CNFs on the surface of PLA retained nanoscale morphology. To prepare for SEM analysis, dry samples were scattered onto carbon-tape-coated SEM stages, and then sputter coating (gold/palladium, 4 nm) was used to promote conductivity. A Zeiss NVision 40 (Oberkochen, Germany) at 3 kV (electron high tension) was used for SEM imaging.

### 2.2. Processing and Testing of Composites

PLA-CNF mixtures were manufactured at 10, 20, and 30 wt.% loading levels by masterbatching the dried PLA-50 wt.% CNF mixture. For masterbatching, powdered PLA was added to PLA-50 wt.% CNF until the target wt.% of CNFs was obtained. Conditioning of the masterbatched materials (>4 h, 50–55 °C) was used to remove residual moisture, after which they were compounded in a Brabender counter-rotating twin-screw extruder (Brabender, South Hackensack, NJ, USA). The compounder temperature was 230 °C, with a screw speed of 60 rpm. The loading zone temperature was set to 160 °C, as higher temperatures caused the fine PLA powder to sinter together before being pulled into the screws, which caused compounding issues. Pure PLA powder was compounded as the control. For PLA-CNF mixtures at lower loading levels (0.5–2 wt.% CNFs, 20 wt.% solids in dewatering), materials were conditioned like the masterbatched samples, after which compounding was performed at 175 °C (60 rpm) in a Brabender 30CC Half Size Mixer (Brabender, South Hackensack, NJ, USA). The smaller mixer was used to accommodate the smaller mixture sizes produced by the dewatering experiments at lower loading levels. For mechanical property comparison between dewatered PLA-CNF and conventional PLA/SDCNF composites, conditioned SDCNFs and powdered PLA were mixed by hand to match compounded PLA-CNF samples, then compounded in the half-size mixer as above. The SDCNFs (90% fines) were provided by the UMaine Forest Bioproducts Research Institute and made by diluting a 3 wt.% CNF slurry to 1 wt.% solids, then drying at 125 °C with a 30,000 rpm atomizer.

After shear-mixing, composites (masterbatched and low-level CNF) were converted to pellets with a plastic granulator (U.S. screen size #6) and stored overnight in an oven (~50–55 °C) to condition pellets for injection molding. Injection molding (Electro-Matic, Inc., Farmington Hills, MI, USA) at 190 °C was used to convert composite pellets into specimens for ASTM D-790 [22] (flexure) and D-7205 [23] (tensile) standard testing. Due to low sample quantities, only standard tensile specimens were made for PLA-CNF composites at 0.5–2 wt.% CNF loading.

To make PLA-CNF films, mixtures of powdered PLA with 0.1–10 wt.% CNFs at 20 wt.% initial mix solids were made and dewatered as above, after which the material cake was directly transported to the oven and dried for 16 h at 50 °C. Aluminum foil was folded until a 0.7 mm thick square was achieved to provide a mold for thin composite films. A square (50 × 50 mm^2^) was cut from the folded sheet to provide the template for the final films. For each mixture, 2 g pieces of sample were added to the square cutout in the mold, which was then assembled between two Teflon caul sheets. The assembly was hot pressed (Carver press, Wabash, IN, USA) at 185 °C and at a pressure of 1.07 MPa, after which a 3 kg weight was used to prevent sample warping during cooling. A Muse 3D Desktop Laser Cutter (Full Spectrum Laser, Las Vegas, NV, USA) was used to cut the square films into strips (50 × 5 mm^2^).

Conditioning at 23 ± 2 °C with 50 ± 2% humidity (RH) for a minimum of 48 h was performed for all specimens prior to mechanical testing. For each sample type, 5 specimens underwent 3-point bending (500 N load cell with a crosshead speed of 1.82 mm/min) and tensile (100 kN load cell with a test rate of 5 mm/min) testing using Instron model #5942 and Instron model #5966 universal test frames (Instron, Norwood, MA, USA), respectively. The greatest tensile strength observed during testing, or ”ultimate tensile strength”, was reported as the tensile testing result. To perform impact testing, 5 pieces of tested specimens from flexural testing per sample type were cut and notched according to ASTM D-256 [24] and conditioned like the tensile specimens. A pendulum impact testing apparatus (CEAST, Instron, Norwood, MA, USA) with a 50 N hammer was used to obtain impact testing results. To understand properties of the compression molded films, the laser cut strips underwent tensile testing in an Instron model #5942 after conditioning similarly to the shear-mixed samples. A crosshead speed of 1 mm/min and gauge length of 35 mm were used. The tensile testing strategy was adapted from a different ASTM standard typically used in thin plastic sheet testing (ASTM D882) [25], albeit with a gauge length below the standard recommendation (between 100 and 250 mm). Typical ASTM standards for tensile testing of sheeting and film specimens (such as ASTM D6287) [26] do not include laser cutting as a standard method for preparing specimens, so this testing method does not have a strong precedent in the literature. Since no uniform standards exist for testing brittle nanocomposite films, this method was only used as a “first pass” to estimate how dewatered CNF loading affected tensile strength before scaling up to larger-scale shear-mixed specimens. An extensometer was not used during thin-film testing due to the small specimen sizes, and therefore strain and modulus were not reported. Statistical significance of differences between properties of the different composites was determined through a multivariate ANOVA (analysis of variance), with an assumed confidence level of 95%. Subgroups of samples that exhibited significant differences were compared using Waller–Duncan post hoc analysis.

### 2.3. Calculations for CNF Monolayer Formation

If CNFs in a well-mixed suspension with PLA particles are at a high enough concentration, the potential for CNF-CNF interaction and agglomeration during drying is assumed to increase. Based on SEM results, it was considered useful to calculate the minimum CNF loading level at which they would preferably interact and form agglomerated sheets around individual PLA particles during drying. These model calculations were performed to promote the understanding of how to optimize dewatered PLA-CNF systems for producing dry fibrillar CNFs without agglomeration, as dewatering below the threshold for the monolayer formation may optimize the “trapping” of individual CNFs between PLA particles to produce well-dispersed, dry fibers with nanoscale dimensions. To construct the model, a spherical particle of PLA was assumed to be 1 µm in diameter, after which its surface area and volume were calculated. Additionally, 1 μm was used to create a baseline for the model due to most of the powdered PLA particles having particle diameters below 150 µm. An assumed PLA density of 1.24 g/cm^3^ was based on the manufacturer specification sheet received for the PLA pellets used in this work. The mass per PLA particle in grams was calculated using density and volume to determine the number of particles in a gram of PLA powder. CNF film density was assumed to be 1.2 g/cm^3^ and multiplied by the theoretical volume of a CNF monolayer of a given thickness covering the PLA particle to obtain the mass of a CNF monolayer per particle. This was multiplied by the number of particles per gram for the assumed PLA particle diameter to obtain the total grams of monolayer-forming CNFs in a gram of PLA, which was multiplied by 100 to obtain the wt.% of CNFs in a PLA-CNF mixture required to form a monolayer. This process was performed for multiple assumed PLA particle diameters (1–100 μm) and CNF monolayer thicknesses (100, 200, 400 nm).

### 2.4. Analysis of Pressed Films with Polarized Light Microscopy

The CNF morphology after compounding and the quality of particle dispersion were evaluated by pressing thin films and analyzing them with polarized light microscopy (PLM). After tensile testing, two hundred milligram pieces were cut from tested specimens. A Caver press was heated to 185 °C, and the specimen pieces were placed between Teflon caul plates and then into the press. The pieces were softened by allowing them to sit in the press for 2 min to ensure the Teflon layer would not be damaged in the pressing event. Pressing was performed at 2.5 MPa for a time of 20 s, and the materials were removed and compressed with a 3 kg weight during cooling. A HaYear computer microscope (0.5× objective, Shenzhen, China) was used for imaging. Cross-polarizers with an additional full wave (red) filter were used, and the semi-crystalline and oriented CNFs exhibited birefringent colors, allowing them to be differentiated from the non-oriented, highly amorphous PLA matrix [27]. A microscope with higher magnification capabilities was necessary to examine lengths, widths, and aspect ratios of the dry fibers in the pressed films, so an Amscope model ME520TA microscope (Irvine, CA, USA) was used. A similar polarization setup was used for PLM, along with a 10× objective lens. Scale bars in the software were set to 50 µm, and at least three images were taken for each sample. A maximum magnification of 10× was used as higher magnifications like 20× only allowed 3–5 fibers to be visualized at a time.

### 2.5. Particle Size Analysis with ImageJ

**Figure 2:**
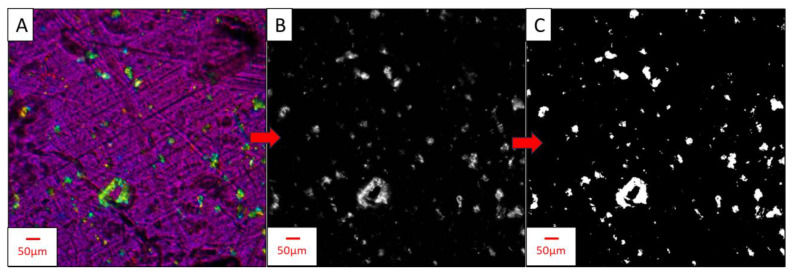
PLM image processing for particle analysis of CNF in PLA-CNF films. (**A**–**C**) show the steps used to pre-process images so that particles can be analyzed by Image J. (**A**) Image of PLA-0.5% SDCNF, cropped to 8 × 8 mm^2^ with scale bar removed. CNF particles are yellow-blue-green, PLA background is magenta-purple. (**B**) “Green” channel from R/G/B channel split. (**C**) Final binary image used in particle size analysis. Scale bar size = 50 μm.

To estimate particle size distributions of CNFs within films made from the shear-mixed samples (0.5–2 wt.% of samples with dewatered CNFs and SDCNFs), PLM images from the previous step underwent particle size analysis with ImageJ [28]. The Amscope images were first imported into ImageJ; then, the measurement scale was set using the on-screen 50 µm scale bar. An 8 × 8 mm^2^ square was cropped out of each image, and the channels were split into R/G/B color channels. A green color channel allowed the birefringent CNFs to be visualized in a binary form. The images were converted into a binary black/white color scheme, and the binary color threshold was set to 54. This process is shown in Figure 2.

An elliptical fit model was used to estimate the major axis, or “length”, and minor axis, or “width”, of the dry CNFs dispersed in the composite films. The elliptical fit model was applied by checking “area” and “fit ellipse” options under “set measurements” in the “analyze” toolbar, then analyzing particles from 0-infinity µm^2^. This was performed for three PLM images per sample type. The resulting data from all three images were combined into a single dataset for each sample type. Aspect ratios were calculated by dividing the major axis by the minor axis for each particle analyzed. All datasets were randomized and scrubbed so that each contained exactly 1982 data points to ensure equal population sizes for each dataset. Averages and histograms of major axes and aspect ratios were obtained using Sigmaplot ver. 13. SPSS software ver. 28 with Shapiro–Wilk and Kolmogorov–Smirnov analyses was used to perform normality testing. Aspect ratios were measured due to being predictors of composite performance based on percolation theory, in which fibers in a composite matrix are theorized to transition to an “infinite” communicating state upon reaching a given percolation threshold [29] (Equation (1)):

(1) $$V_{Rc}=\frac{0.7}{L/d}.$$

where $V_{Rc}$ is the volume fraction of fibers at which they will form this communicating state, and L/d is length over diameter or aspect ratio of the fibers in the composite matrix. Higher aspect ratio fibers decrease the volume fraction of fibers needed to achieve percolation. This communication is theorized to increase mechanical properties of the final composite.

One hundred-gram portions of wet mixed samples were weighed out for water removal calculations. Moisture content was determined for each formulation to establish the starting mass of solids in the samples. Each sample was pressed at least five times using the same parameters as in the initial material manufacture section. The resulting solids after pressing were calculated by comparing the mass recovered to the starting solids of the material. The drying energy requirement per gram of dried CNF for each mixture was determined using a theoretical energy calculation based on the specific heat capacity of water (Equation (2))

(2) q=mcΔT

where q is the total heating energy in joules, m is the mass in grams, c is the heat capacity of water (4.186 J/g × °C), and ΔT is the change in temperature in °C. The latent heat of evaporation for water (2260 J/g) was then multiplied by the mass of water remaining, then added to the total heating energy from Equation (2) to obtain theoretical values for “drying energy”. These results were compared to empirical data for the energy required to produce SDNCF obtained from Oak Ridge National Laboratories. The energy calculations were based on the drying and water removal process, and therefore, energy considerations for cryocrushing PLA to produce powder were not included.

Hard-to-remove water (HRW) analysis was carried out in accordance with previous methods [16,30,31]. Hard-to-remove water content (%/%) was calculated by measuring the total mass percent at the start of the time-dependent second derivative drop in mass, subtracting the final mass percent after drying had completed (percent solids of material), then dividing by the final mass percent (Equation (3)).

(3) $$HRW\;\left(\%/\%\right)=\frac{\left(m_{1}-m_{2}\right)}{m_{2}}.$$

where m_1_ and m_2_ are the mass % at the start of the 2nd derivative drop and the final solids of the material, respectively. This was performed for two samples of PLA-CNF at 2, 10, 30, and 50 wt.% CNF, with the results being reported in terms of averages of the two HRW results.

## 3. Results and Discussion

### 3.1. PLA-CNF Mixture Characterization

The majority of received PLA powder had particle diameters below 250 µm (95.6 wt.%), as shown in sieve analysis results (Table 1). Up to 56 wt.% of the particles were below 150 µm, which was the lowest sieve size used. For dried mixtures with 10 wt.% CNFs, particle diameters increased remarkably, as shown by the fraction of particles with diameters above 250 µm increasing from 4.5 wt.% (pure PLA powder) to 42.7 wt.%. This change was even greater for the PLA-50 wt.% CNF mixtures, in which 85.7 wt.% of the particles were above 250 µm. These results, coupled with the decreasing weight fraction of smaller particles in the samples, indicated that CNF at 10 wt.% and above in mixtures were interacting during drying and forming agglomerates larger than the individual PLA particles used to dewater them.

**Figure 3:**
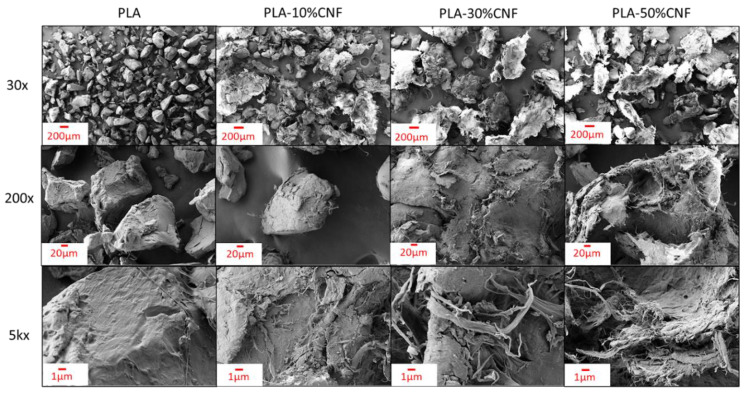
Dried and milled PLA-CNF mixtures under SEM.

From SEM imaging (Figure 3), CNFs can be seen attaching to the surfaces of individual PLA particles and forming agglomerated sheets around them after dewatering and drying in a 10–50 wt.% mixture of PLA and CNFs. Fibrils with nanoscale dimensions are observable, but the dominant morphology of dry CNFs in the mixtures appears to be agglomerated sheets. The milling process used to separate particles after drying is likely the cause of observed individual fibrils, as the fibrillar structure is mostly observed at the ends of agglomerated sheets. Dewatering with 10 wt.% CNFs appeared to reduce agglomeration compared to 30 and 50 wt.% loading levels, with some particles exhibiting lower amounts of attached CNFs. Multiple observed agglomerates appeared to have diameters up to half a millimeter, with multiple PLA particles adhered together in the CNF mass. PLA-50 wt.% CNF was chosen for masterbatching and compounding, as larger CNF agglomerates existed in all dried mixtures above 10 wt.% CNF, and the initial higher loading level allowed for easier control over the CNF concentrations chosen for compounding (10/20/30 wt.% CNFs). It was also theorized that shear mixing could break up CNF agglomerates to provide better fibril morphology in the compounded materials.

### 3.2. Testing of Composites

**Figure 4:**
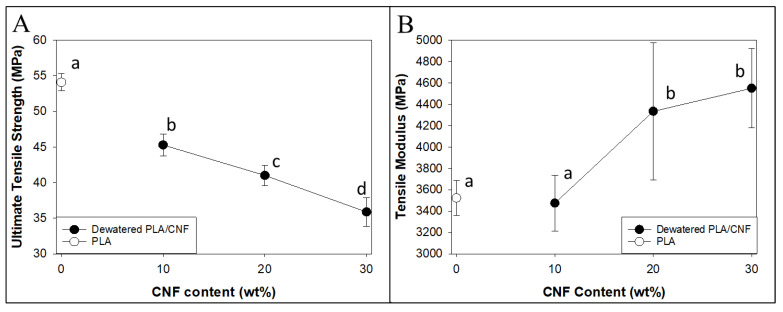
Results for tensile testing of masterbatched PLA-CNF composites and the PLA control. (**A**) Tensile strength. (**B**) Tensile modulus. Common letters on data points indicate statistically insignificant differences at 95% confidence level Error bars represent standard deviation.

Initial mechanical test results from masterbatched PLA-CNF composites can be seen in Figure 4. Composite tensile strength decreased significantly with increasing masterbatched CNF concentration, especially compared to the PLA control. The low aspect ratios, spherical structure, and heterogeneous particle dispersion of the dewatered CNFs at 10–50 wt.% (Figure 3) likely caused poor tensile performance. A significant increase in tensile modulus compared to the PLA control was observed at 20 wt.% CNF loading and above. This showed that while strength decreased, the PLA matrix was still being reinforced by the stiffer dewatered CNF agglomerates. Similar trends were observed in flexural and impact properties of masterbatched composites, where flexural modulus values increased, and strength values decreased with increasing masterbatched CNF ratios (Table S1).

**Figure 5:**
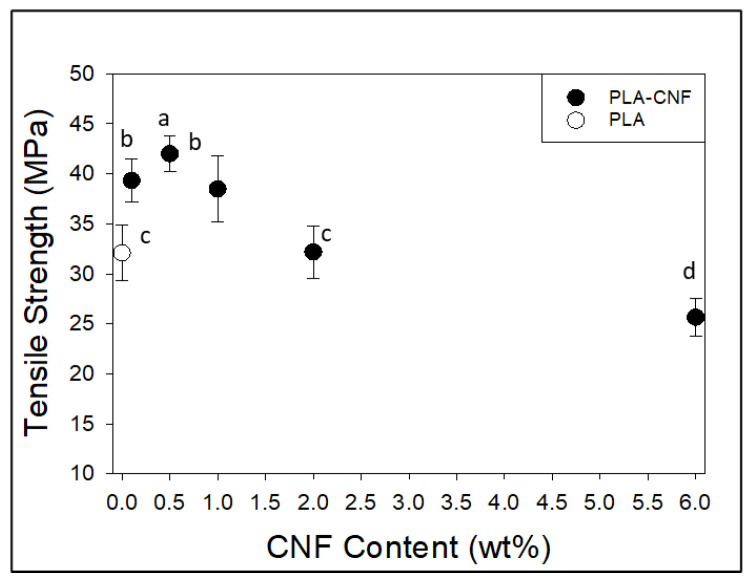
Tensile test results for compression molded PLA-CNF films with respect to CNF loading level. Common letters on data points indicate statistically insignificant differences at 95% confidence level. Error bars represent standard deviation.

At low-level additions, film testing results (Figure 5) show that dewatered CNF loading into hot-pressed films significantly increased film tensile strength compared to pure PLA at 0.1–1 wt.% CNF loading levels, with a reduction in tensile strength above 2 wt.% CNF. The tensile strength values of PLA films (32 MPa) were much lower than those observed from ASTM samples (~54 MPa). The use of laser cutting as a sample preparation method may have led to the low film tensile strength of PLA, as a pulsed laser may have formed serrated edge patterns on cut strips that acted as defect propagation points. If the press time or pressure was not enough to homogenize the properties of thin films from powder PLA, then hot pressing parameters may have also contributed to a weaker thin film strength. A similar result was seen in another work utilizing chitin nanocrystals in a thin film PLA composite, where the control PLA film showed an ultimate tensile strength of 44 MPa [32]. The cutting method for this work was not specified. This does not provide a strong comparison to how injection-molded specimens will perform in tensile testing but can act as a “first pass” method to determine how CNF incorporation affects the tensile properties of PLA. Despite this comparative weakness in PLA, coefficients of variation were low across all sample types (2–9%), indicating that the differences seen between different films can be attributed mostly to CNF loading level.

**Figure 6:**
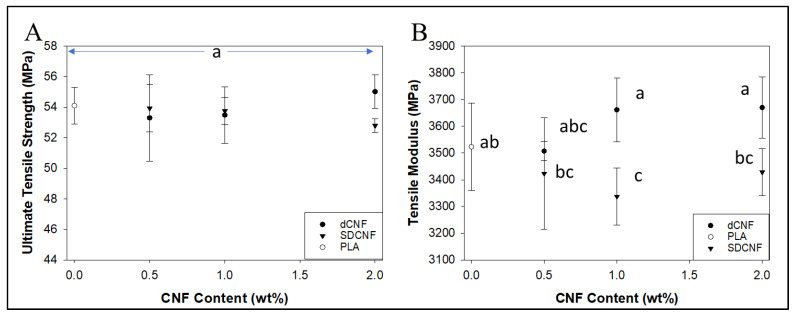
Results for tensile strength (**A**) and tensile modulus (**B**) of shear-mixed dewatered PLA-CNF composites at low loading levels. Common letters on data points indicate statistically insignificant differences at 95% confidence level. Error bars represent standard deviation.

In shear-mixed composites (Figure 6), dewatered CNF (dCNF) did not significantly increase tensile strength or modulus compared to pure PLA up to 2 wt.% dCNF, although a 1.7% increase in average ultimate tensile strength and a 4.2% increase in average tensile modulus compared to pure PLA was observed at a 2 wt.% dCNF loading level. dCNF composites had similar or better ultimate tensile strength and tensile modulus values than SDCNF composites at the same CNF loading levels, which was significantly different at 2 wt.% loading of CNFs. The increased modulus for dewatered samples could indicate better preservation of fibrillar aspect ratios compared to SDCNFs, leading to better composite reinforcement properties. These tensile results are similar to or better than those achieved in other reports that attempted to incorporate SDCNFs into PLA at loading levels below 10% without using modification of CNFs or solvents for dissolution [19,33,34]. More recent work has shown spray-dried CNF significantly improving the performance of PLA/CNF nanocomposites relative to neat PLA via masterbatching methods, and similar methods could potentially be used here to improve results [35].

### 3.3. Modeling for CNF Monolayer Formation

**Figure 7:**
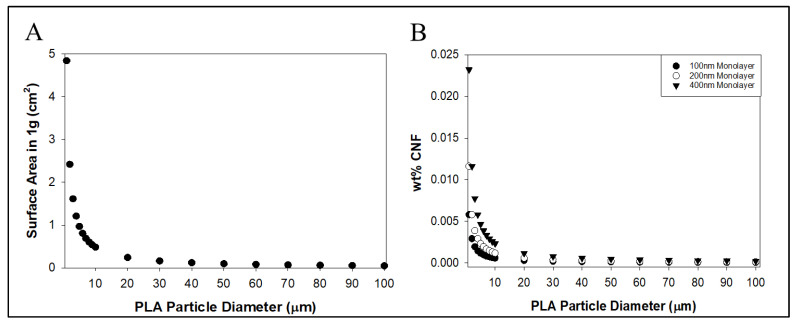
(**A**) Total estimated surface area in 1 g PLA particles over particle diameter. (**B**) Estimated threshold wt.% CNFs in PLA required to form a monolayer over each PLA particle based on PLA particle diameter.

Based on the monolayer formation model presented in the Methods section, a 400 nm thick monolayer of CNFs should form over each individual PLA particle in a PLA-CNF mixture at 0.023 wt.% CNFs assuming a PLA particle diameter of 1 μm (Figure 7B). This value drops to 0.012 and 0.005 wt.% CNFs for 200 and 100 nm thick monolayers, respectively. Below these values, it can be assumed that CNFs remain well-dispersed as fibrils without forming agglomerates around individual PLA particles or bridging them, as seen in the SEM results in Figure 3. Expectedly, the wt.% values for monolayer formation decreased in a power relationship (R^2^ = 1) based on particle diameter, like the relationship between the overall surface area of 1 g of PLA particles and particle diameter (Figure 7A). These calculations indicate that the overall PLA particle surface area influences the tendency of CNFs to form agglomerates over PLA particles rather than remaining dispersed between them. At PLA particle diameters of 20 μm and above, the model begins to asymptote toward zero, indicating vanishingly small amounts of CNF causing agglomeration around PLA particles. This model does not account for the dispersion of CNFs in PLA at varying total mixture weight fractions and can be made more accurate through empirical observation of the PLA-CNF agglomeration process. The model also assumes uniform particle diameters, while the actual particles had highly variable diameters (Table 1). Despite the limitations of this model, the low wt.% CNF agglomeration thresholds observed here helped to inform the decision to lower dCNF loading into PLA to 2 wt.% and below.

### 3.4. Pressed Films with Polarized Light Microscopy Analysis Results

**Figure 8:**
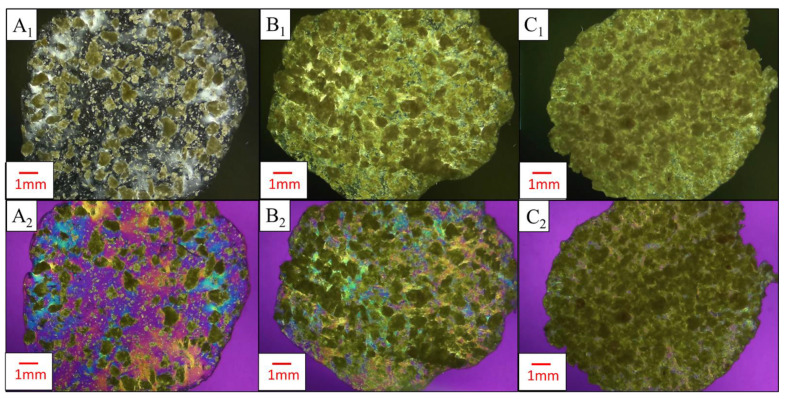
PLA-CNF composite films made from masterbatched PLA-50% CNF visualized with PLM. Letters correspond to CNF loading level, numbers denote microscopy type. (**A**–**C**) 10, 20, 30 wt.% CNF. 1, 2: PLM between crossed polarizers without a retardation filter and PLM between crossed polarizers with a retardation filter, respectively.

Results from polarized light microscopy of masterbatched PLA-CNF films are shown in Figure 8. CNF particles exhibit birefringent blue/yellow/green colors, while the non-birefringent PLA background is purple/magenta when a full-wave retardation filter is used. Thicker agglomerates of CNF do not let much of the light pass and are visually dark green as interference colors are mixed. In the 10 wt.% CNF cross-polarized image obtained with the retardation filter (Figure 8A_2_), blue-yellow film regions with no visible particles were observed in the PLA film, which corresponds to white regions observed in the film with only cross-polarizing filters (Figure 8A_1_). This may be attributed to CNF increasing the crystallinity of the polymer film, therefore providing crystalline orientation that can be observed with PLM, although this has not been confirmed by more accurate methods for determining crystallinity such as differential scanning calorimetry (DSC). The morphology of CNF in the films is dominated by spherical agglomerates up to a millimeter in diameter. Smaller and higher aspect ratio particles are observed in the PLA-10% CNF film (Figure 8A_2_). As CNF loading is increased, the dispersion of the CNF agglomerates is reduced, and particles contact each other more frequently within the PLA. This “crowding” effect could have caused the increased moduli and decreased strength of the composites seen in Figure 4. The moduli increased attributable to the higher stiffness of interacting CNF particles in the PLA composite, but the mechanical strength decreased because of poor dispersion of the crowding PLA particles and the large size and low aspect ratios preventing the percolation effect for reinforcement. These results, along with the SEM images in Figure 3, showed that PLA particles did not effectively “trap” CNFs to prevent agglomeration at 10–50 wt.% loading levels. This observation was instrumental in the decision to lower the CNF loading levels to determine the threshold at which CNFs would be effectively “trapped” by PLA particles during dewatering and drying.

**Figure 9:**
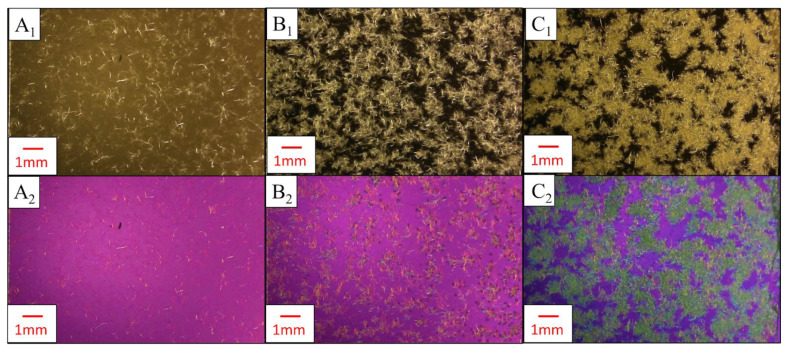
Polarized light microscopy images of PLA-CNF composites made at lower CNF loading levels. Letters correspond to CNF loading level; numbers denote microscopy type. (**A**–**C**) 0.5, 2, 6 wt.% CNF. (**1**,**2**): PLM between crossed polarizers without a retardation filter and PLM between crossed polarizers with a retardation filter, respectively.

At lower CNF loading levels, especially 2 wt.% and below, dewatering appears to preserve fibril dimensions after drying (Figure 9). Well-dispersed fibrils were observed in films with 0.5 wt.% CNFs, and lightly agglomerated networks appeared at 2 wt.% CNFs. This is likely around the threshold at which the PLA particles are capable of trapping and dispersing CNF particles to prevent agglomeration, shown by the reduction of film mechanical properties at 2 wt.% CNF in Figure 5. This agglomeration increases with higher CNF loading, shown by the heterogeneously dispersed millimeter-scale structures of CNFs observed in PLA-6 wt.% CNF films (Figure 9C_2_), along with the further decrease in film tensile strength at this loading level.

**Figure 10:**
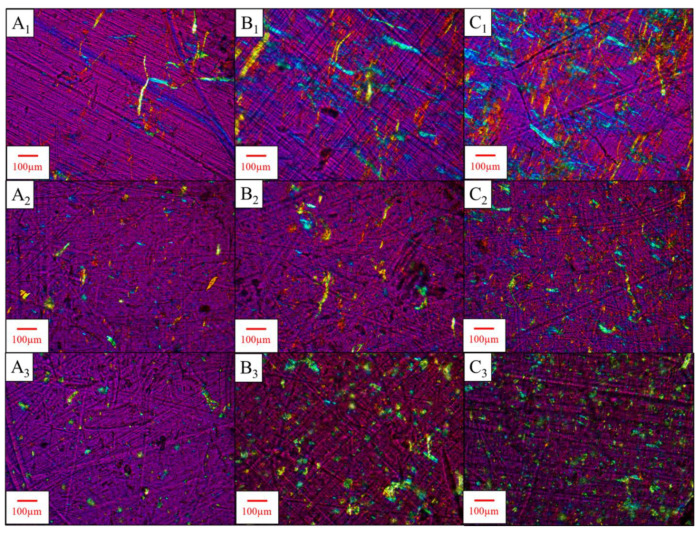
PLM images of PLA-CNF films at lower loading levels of CNF. Letters correspond to CNF loading level, numbers correspond to processing/CNF type. (**A**–**C**) 0.5, 1, 2 wt.% CNF. 1, 2, 3: compression molded PLA-dCNF films, shear-mixed PLA-dCNF, and shear-mixed PLA-SDCNF. Objective lens: 10×.

In Figure 10, reddish fibril networks can be observed in the non-compounded dewatered PLA-2 wt.% dCNF film (Figure 10C_1_), which can be attributed to CNF fibrils that are out of phase with the polarized light beam passing through the sample. CNF fibers with maximum widths approaching 45 μm for dewatered CNF and 70 μm for SDCNF were observed, with most particles appearing to be tens of microns in width. Shear mixing reduced the overall fibril lengths and broke up loosely associated fibril networks in PLA-2 wt.% dCNF to produce dispersed CNF fibrils with similar dimensions to PLA-0.5/1 wt.% dCNF. This could explain the differences in mechanical properties between dewatered CNF-loaded samples with and without shear mixing, as the agglomerated networks in the PLA-2 wt.% dCNF film that may have reduced tensile strength were converted into loosely percolating fibrils that slightly increased the tensile strength and stiffness of the final shear-mixed composite. Since PLA-2 wt.% dCNF was the maximum loading level tested here and showed better relative properties to PLA in shear-mixed samples than in films because of this fibril breakup and dispersion during shear mixing, higher levels of CNF loading may also produce similar effects after shear mixing. The maximum loading threshold at which CNF can be dewatered and shear-mixed without reducing the tensile properties of the final composite has not been determined.

Large fibers in compression molded films (Figure 10A_1_–C_1_) were difficult to measure utilizing ImageJ ver. 1.53s software macros, so manual measurements of individual fibers were used. Fibers between tens and hundreds of microns in width and hundreds of microns in length were measured. While nanoscale dimensions were not directly measurable at the resolution available with optical microscopy, high aspect ratios of up to 112 were calculated. High aspect ratios may have decreased the composite percolation threshold and result in the highest film tensile properties at 0.1–1 wt.% loading levels. Overall, the dewatered CNF appeared to have more fibrous structures and fewer spherical forms than SDCNF, which may explain the higher tensile properties at 1–2 wt.% CNF loading in shear-mixed composite testing. Since agglomeration is more clearly seen at 2 wt.% dCNF and above, where film tensile strength begins to decrease, this is likely closer to the “monolayer formation” or agglomeration threshold at 20 wt.% mixing solids. This indicates that the model from the monolayer formation section that shows CNFs forming spherical monolayers around PLA particles at 0.025 wt.% and below needs to be adjusted to properly account for how CNFs agglomerate in PLA-CNF mixtures.

### 3.5. Particle Size Analysis Using ImageJ

**Figure 11:**
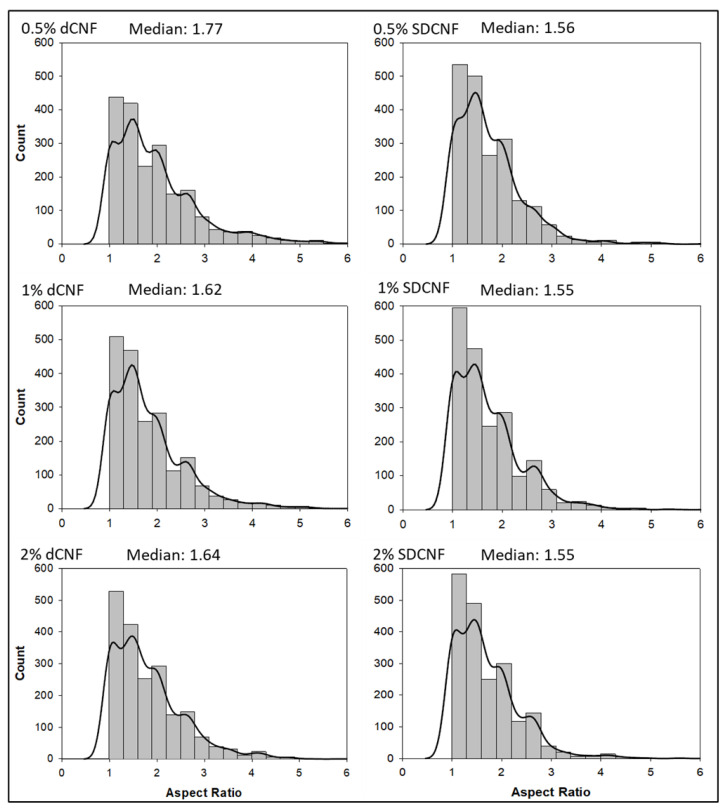
Aspect ratio histograms from analysis of PLA-dCNF and PLA-SDCNF PLM images. Bin size = 0.333 μm/μm. Bandwidth for kernel density overlay= 1.507 × 10^−1^. Median aspect ratios are listed above each distribution.

Aspect ratio distributions from ImageJ analysis (Figure 11) of PLM images from shear-mixed samples show that most measured particles had aspect ratios between 1 and 5. All distributions have high positive skew (>2), and most measured particles had aspect ratios below 2. All SDCNF composites showed a higher count of particles with aspect ratios below 2, especially between 1 and 1.7. Shapiro–Wilk and Kolmogorov–Smirnov tests rejected the normality of all datasets, indicating that averages, standard deviations, and standard hypothesis testing to quantify differences between datasets may be unreliable. Medians of all datasets were therefore reported, with all dewatered CNF composites showing higher median dCNF aspect ratios than SDCNF composites. The higher dCNF aspect ratios may have contributed to the higher mechanical properties seen with dCNF compared to SDCNF at similar loading levels (Figure 6), even if the quantified differences among datasets are small. The analysis used an elliptical fit model, which may have caused issues in accurately measuring the length of longer fibrils with variable widths and orientations. The maximum measured aspect ratio range for dCNF datasets (8.3 for PLA-2 wt.% dCNF to 16.7 for PLA-0.5 wt.% dCNF) was higher than that of SDCNF datasets (6.2 to 10.1). Dry fibrils at least hundreds of nanometers in width were likely preserved after drying, and fibrils with even smaller widths may exist but not have been detected by the imaging system. The wavelength of visible light is between 380 and 700 nm; therefore, fibers below 380 nm in width should not have been measured using this method. In addition, only using the “green” color channel may have prevented the software from detecting particle dimensions with other birefringence colors, such as blue, and, therefore, reduced the overall measured particle size. Even with these potential limitations in measurement, this can be used as a first-pass method to indicate how aspect ratios of dewatered CNF compare to spray-dried CNF, as shown by the different median aspect ratios between the two groups and the relatively similar median aspect ratios within the SDCNF measurements.

### 3.6. Contact Dewatering Water Removal and Energy Analysis

**Figure 12:**
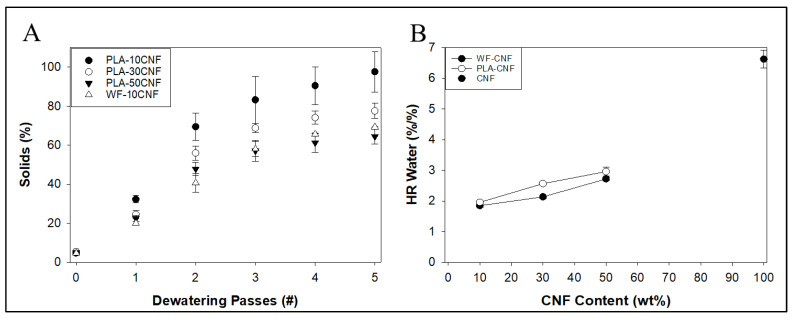
(**A**) Percent solids of PLA-CNF mixtures after multiple dewatering passes, compared to WF-10% CNF. (**B**) HR water contents of PLA-CNF and WF-CNF furnishes (CNFs at ~3% solids, mixtures at ~5% solids).

As seen in Figure 12A, water removal shown by final wt.% solids was calculated based on the starting moisture content and mass of the pressed mixture from 10 to 50 wt.%. PLA-CNF seems to follow a polynomial relationship with number of presses (R^2^ = 0.98–0.99), which begins to asymptote for PLA-10% CNF, PLA-30%, and PLA-50% CNF after 4–5 presses (80–99 wt.% solids, 74 wt.% solids, and 60 wt.% solids, respectively). Water removal for PLA-CNF mixtures was much higher than that of WF-CNF furnishes from the previous work [16] at similar loading levels, possibly due to the smaller PLA particle size, with 95.6 wt.% and 23 wt.% of PLA particles and maple WF fibers used in that work, respectively, having diameters below 250 µm (Table 1). The smaller size of PLA particles and, therefore, higher surface area for solid PLA to contact wet CNF fibrils likely caused more efficient dewatering. Wood flour can also hold approximately 30% of its dry weight in water (70 wt.% solids at fiber saturation point or FSP), while water saturation capacity for less porous, less hydrophilic PLA is likely a lot lower. PLA-10% CNF solids had much higher standard deviations than all other mixtures. At 5 wt.% solids, the PLA-10 wt.% CNF mixture was much less viscous than all others, which may have led to material loss during pressing and, therefore, discrepancies in dewatering results. HR water results (Figure 12B) show that, while water removal was higher using PLA to dewater CNF compared to WF, this does not correlate to different levels of tightly bound water between the dewatering material types, as PLA-CNF mixtures had slightly higher HR water contents than WF-CNF furnishes at similar loading levels.

**Figure 13:**
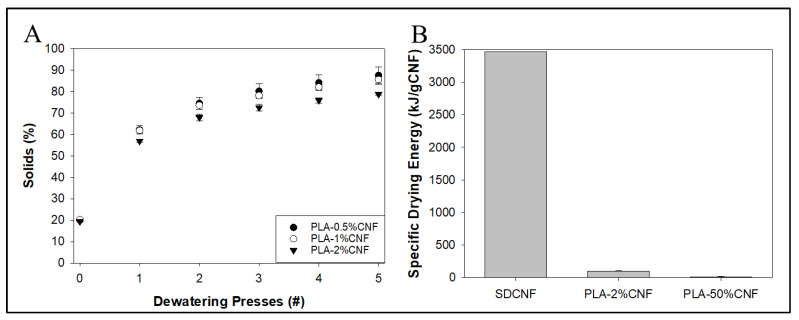
(**A**) Percent solids of low CNF mixtures after multiple dewatering passes. (**B**) Theoretical specific drying energy per gram of CNFs calculated for PLA-2%dCNF (20 wt.% starting solids) and PLA-50% dCNF (5 wt.% starting solids) after 1× dewatering passes compared to 1× pressed CNFs.

Similar properties were seen with dewatering lower levels of CNF (Figure 13A) at 20 wt.% solids, with solids content showing asymptotes around 4–5 presses (79 wt.% solids for PLA-2% CNF, 86 wt.% solids for PLA-1% CNF, and 87 wt.% solids for PLA-0.5% CNF). The solids content increased more after one press (from 20 to ~60 wt.% solids) for all low-level CNF mixtures compared to the high-level CNF mixtures (from 5 to ~30 wt.% solids), likely due to the higher solids content facilitating water removal from the system. The solids content asymptote is around 79–87%, and the low standard deviation confirms the previous observation that this method may be able to achieve 80–90% solids for dewatered CNF mixtures. This may also correlate with the amount of water retained by PLA itself, as the increase in water removal, as shown by solids content, is negligible between 0.5 and 1 wt.% CNF. Measurement of water removal may have been affected by water re-absorption by the dewatered cake, potentially making measurements imprecise.

Theoretical energy required to dry the system per gram of “dry” CNF (Figure 13B) based only on the amount of remaining water in the sample was more favorable for dewatered CNF than for empirical values obtained from the Oak Ridge National Laboratories data on the spray-drying process. Compared to the 3465.2 kJ/gCNF for SDCNF, our PLA-2 wt.% CNF system showed a theoretical drying energy of 101.3 kJ/gCNF after just one dewatering pass or a 34-fold reduction in energy use. PLA-50% CNF showed a theoretical drying energy of 17.8 kJ/gCNF after one dewatering pass or a 195-fold reduction in energy use compared to SDCNF, but the unfavorable mechanical properties shown from masterbatching PLA-50% CNF may negate any advantage in energy savings. PLA-2%dCNF showed a theoretical dewatering energy of 35.8 kJ/g after five presses. The theoretical drying energy calculations were made with an assumption of heating water from 25 °C to 100 °C, whereas the actual materials were dried at 50–55 °C. This would likely cause differences between theoretical and actual drying energy requirements of the material of the power use of an oven or similar drying method that was empirically measured.

## 4. Conclusions

CNFs were directly dewatered using PLA for composite applications, which no other research had attempted before. Dewatered CNFs were successfully incorporated into shear-mixed PLA/CNF composites while positively affecting mechanical properties after optimizing dewatering to produce micron-nanoscale dry cellulose fibers, with results that were as least as good as spray-dried CNFs in PLA. The following conclusions were drawn from the results and analyses presented in this work:

- Dewatered CNF at loading levels of 0.1–2 wt.% in PLA powder preserved nano-to-microscale CNF dimensions in pressed PLA films;
- In compression molded films, CNFs significantly increased the tensile properties of PLA in the range of 0.1–1 wt.% loading, but agglomeration at 2 wt.% and above reduced tensile strength compared to pure PLA;
- In shear-mixed composites, PLA with dewatered CNFs showed comparable tensile strength and better tensile modulus than pure PLA or PLA with SDCNF at 1–2 wt.% dewatered CNF loading levels;
- Modeling the agglomeration threshold based on spherical particles did not agree with results obtained from PLM image analysis, so the model assumptions should be updated to more accurately reflect the fibril agglomeration;
- Shear-mixing PLA-2 wt.% dCNF broke up agglomerates seen in compression molded films and produced test samples with the highest tensile properties;
- In subsequent work, higher loading levels of PLA-dCNF could be shear-mixed and tested to determine the maximum level of CNFs that can be dewatered onto PLA without reducing mechanical properties;
- Dewatering is a much more energy-efficient method of producing dried, micron-to-nanoscale CNFs for composites than spray-drying.

## Figures and Tables

**Table 1:** Results from sieve analysis for PLA particles and PLA-CNF mixtures after dewatering, drying, and milling.

Mixture	Fraction Diameter (μm)	Mass %
PLA	>250	4.5
	150–250	39.2
	<150	56.3
PLA-10% CNF	>250	42.7
	150–250	36.8
	<150	20.5
PLA-50% CNF	>250	85.7
	150–250	8.7
	<150	5.6

## Data Availability

Data is contained within the article.

## Supplementary Materials

The following supporting information can be downloaded at https://www.mdpi.com/article/10.3390/nano14171419/s1, Table S1: Summary of average shear-mixed PLA-CNF composite properties (with standard deviations) from tensile, and notched IZOD impact testing. Figure S1: Histograms of major axis dimensions (bin size = 0.333 μm) from particle size analysis of PLM images with kernel density overlays, (bandwidth = 4.453 × 10^−1^).

## Abbreviations

Cellulose Nanofibrils (CNFs), Poly(lactic acid) (PLA), Wood Flour (WF), Spray-dried CNFs (SDCNFs).
