# Supplementary material for: Cellulose Nanofibrils Dewatered with Poly(Lactic Acid) for Improved Bio-Polymer Nanocomposite Processing
Source: Nanomaterials (Basel). 2024 Aug 30;14(17):1419. doi: 10.3390/nano14171419 (PMC11397092; doi:10.3390/nano14171419)
Supplement: Supplementary file 1 [file nanomaterials-14-01419-s001.zip › nanomaterials-3084707-supplementary.pdf]

Cellulose Nanofibrils Dewatered with PLA for Improved Bio-polymer Nanocomposite Processing

Alexander Collins, Mehdi Tajvidi*

School of Forest Resources ad Advance Structures and Composites Center, University of Maine, Orono, ME, USA, 04469

*corresponding author: mehdi.tajvidi@maine.edu

Supplementary Materials

Table S1: Summary of average shear-mixed PLA-CNF composite properties (with standard deviations) from tensile, and notched IZOD impact testing

		Tensile		Flexural		Impact
**Composite**	**CNF Type**	**Strength (MPa)**	**Modulus (GPa)**	**Strength (MPa)**	**Modulus (GPa)**	**Strength (J/m)**
PLA	N/A	54.1 ± 1.2	3.52 ± 0.16	N/A	N/A	N/A
PLA-0.5%	dCNF	53.3 ± 2.8	3.51 ± 0.04	N/A	N/A	N/A
PLA-1%	dCNF	53.5 ± 1.8	3.66 ± 0.12	N/A	N/A	N/A
PLA-2%	dCNF	55.0 ± 1.1	3.67 ± 0.11	N/A	N/A	N/A
PLA-10%	dCNF*	45.3 ± 1.5	3.48 ± 0.26	77.3 ± 4.2	3.16 ± 0.25	26.6 ± 2.1
PLA-20%	dCNF*	41.0 ± 1.5	4.33 ± 0.64	71.8 ± 2.7	3.84 ± 0.28	28.8 ± 6.2
PLA-30%	dCNF*	35.9 ± 2.0	4.55 ± 0.37	57.0 ± 5.8	4.26 ± 0.37	18.5 ± 3.8
PLA-0.5%	SDCNF	53.9 ± 1.6	3.42 ± 0.21	N/A	N/A	N/A
PLA-1%	SDCNF	53.7 ± 0.9	3.34 ± 0.11	N/A	N/A	N/A
PLA-2%	SDCNF	52.8 ± 0.5	3.43 ± 0.09	N/A	N/A	N/A

Abbreviations: PLA = poly(lactic acid), dCNF = dewatered CNFs, SDCNF = spray dried CNFs.

**** From formulations masterbatched at PLA-50wt%CNF***

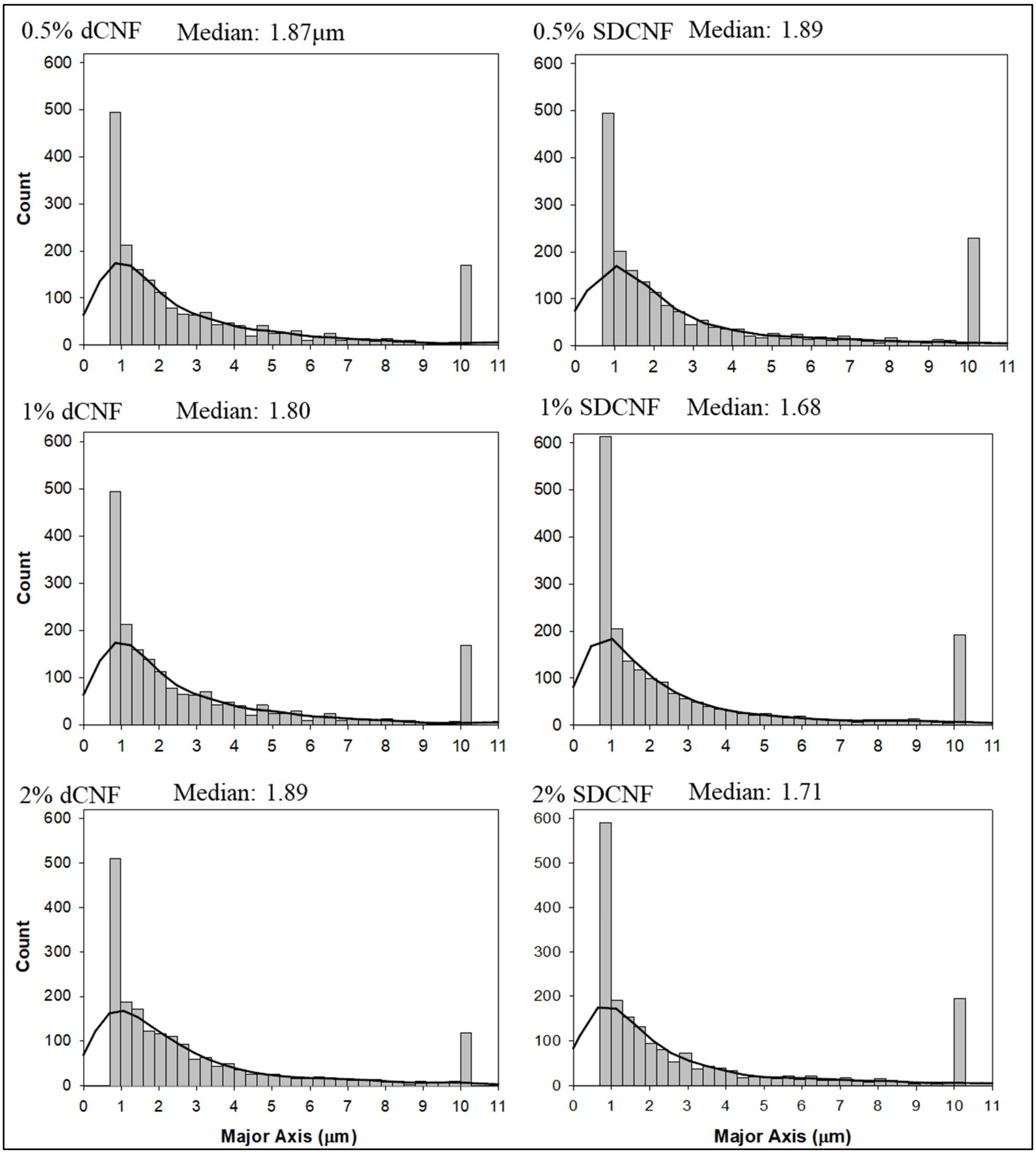

Figure S1: Histograms of major axis dimensions (bin size = 0.333 µm) from particle size analysis of PLM images with kernel density overlays, (bandwidth = $4.453*10^{-1}$).
